# The Studies on Chitosan for Sustainable Development: A Bibliometric Analysis

**DOI:** 10.3390/ma16072857

**Published:** 2023-04-03

**Authors:** Weng Siew Lam, Weng Hoe Lam, Pei Fun Lee

**Affiliations:** Department of Physical and Mathematical Science, Faculty of Science, Universiti Tunku Abdul Rahman, Kampar Campus, Jalan Universiti, Bandar Barat, Kampar 31900, Perak, Malaysia; lamws@utar.edu.my (W.S.L.); pflee@utar.edu.my (P.F.L.)

**Keywords:** chitosan, bibliometric analysis, biopolymer, sustainability, VOSviewer

## Abstract

Chitosan is a biocompatible polymer with vast applications in pharmacology, medicine, paper making, agriculture, and the food industry due to its low toxicity. Chitosan also plays an important role in the sustainable environment since chitosan is able to absorb greenhouse gases, harmful organic matter, and heavy ions. Therefore, this paper conducts a bibliometric analysis of chitosan for sustainable development using the Scopus database from 1976 to 2023. A performance analysis on the 8002 documents was performed with Harzing’s Publish or Perish. Science mapping was conducted using VOSviewer. The annual publication on chitosan for sustainable development showed an upward trend in recent years as the annual publication peaked in 2022 with 1178 documents with most of the documents being articles and published in journals. Material science, chemistry, and engineering are tightly related subject areas. China had the highest publication of 1560 total documents while the United States had the most impactful publication with 55,019 total citations, 68.77 citations per document, 77.6 citations per cited document, *h*-index 110, and *g*-index of 211. India had the largest international collaboration with 572 total link strength. “International Journal of Biological Macromolecules”, “Carbohydrate Polymers”, and “Polymers” have been identified as the top three source titles that publish the most documents on chitosan for sustainable development. The emerging trends in chitosan on sustainable development focus on the application of chitosan as an antibacterial agent and biosorbent for contaminants, especially in water treatment.

## 1. Introduction

Derived from chitin, chitosan is a polysaccharide produced from the skeletons of crustaceans or the walls of fungi [1]. Chitin (β-(1→4)-*N*-acetyl-D-glucosamine) is abundantly available, however, its application is less favored due to its hydrophobic nature [2]. Pure chitin is translucent, resilient, and tough. Chitin exists in the α and β allomorphs. α-Chitin is compact with strong hydrogen bonding while β-chitin has weak intermolecular hydrogen bonding. Chitin, which is white and hard, can be obtained naturally as ordered crystalline microfibrils. Through deacetylation, chitosan, which is a β-1,4-D-glucosamine, can be derived from chitin, an *N*-acetyl-D-glucosamine [3,4]. Since chitosan is obtained from renewable resources, it is biodegradable with a low toxicity. The United States, Europe, Korea, and Japan have granted approval for the use of chitosan for consumption [5]. Moreover, when the amino groups in chitosan undergo protonation, chitosan becomes cationic and hydrophilic in an acidic aqueous solution, which is of great interest for biomedical and pharmaceutical applications [6]. This is contributed by the presence of a free amino group in chitosan, which is absent in chitin. Chitosan is easily available and relatively cheap with good biocompatibility, biodegradability, and ease of chemical modification.

Chitosan has a high degree of deacetylation (DD), which is normally above 40%. Hence, its solubility, crystallinity, and antimicrobial activity can be modified through its reactive sites such as the hydroxy (-OH) and amino (NH_2_) groups to suit various applications [7]. Chitosan, with amino acid modification, is biocompatible for wound healing and tissue generation [8,9]. Chitosan can also be modified for antibacterial and antifungal properties to inhibit the growth of *E. coli* and *B. cinerea* [10]. Chitosan-based sprays and aerogels provide a significant reduction in bacterial growth and are good for food packaging [11,12]. Amino acid-modified chitosan with folic acid is also hemocompatible to target cancer cells and decrease tumor spheroid volume [13].

Quaternized chitosans serve as absorbents to capture higher uptakes of greenhouse gases [14]. In addition, phosphorylated chitosan can be used as a flame retardant and is highly investigated for heat insulation [15,16]. Carboxymethyl chitosan is also favored in the cosmetics industry as a source of antioxidants for hydration and protection of the skin [17]. Moreover, chitosan serves as a scaffolding material for cell growth and tissue engineering, as the cationic property of chitosan allows interaction with glycoproteins and other structural molecules [18]. Chitosan is also used as a stabilizer in emulsions for the food industry [19].

Based on the concept of circular economy and the United Nation’s Sustainable Development Goals (SDGs), companies are encouraged to always consider the present and the future in their production and consumption. Chitosan is a biopolymer that contributes to sustainability in industry. Chitosan can replace harmful chemicals in agriculture while protecting crops from diseases due to its antimicrobial activity [20,21]. Chitosan composites are also good absorbents of pollutants and metal ions such as tartrazine, tetracycline hydrochloride, arsenic, uranium, Pb^2+^, Cu^2+^, and Hg^2+^ ions [22,23,24,25,26]. Chitosan can also be used to manufacture paper so that the use of chemical additives can be reduced [27,28]. Moreover, chitosan helps to capture harmful gases such as formaldehyde and greenhouse gases [29,30].

One great advantage of chitosan is that chitosan can act as a biocompatible and biodegradable substrate for electronics. A maleic–chitosan proton conducting layer has good field-effect proton mobility and is a breakthrough discovery in green electronics [31]. Starch–chitosan substrate-based transparent electrodes can also be used in wearable electronics [32]. Silver nanowire–chitosan substrate can be used as the bottom electrode for perovskite solar cells with good stability [33]. Optimized chitosan electrostatic layer-by-layer films acting as cathode interlayers for inverted organic solar cells show high power conversion efficiency and could reduce the work function of electrodes to improve device performances [34]. Chitosan with yttrium oxide (Y_2_O_3_) can act as gate dielectric thin films in organic thin-film transistors with improved dielectric characteristics and pinholes and could operate in low voltage situations for various curvature radii [35].

Based on the vast applications of chitosan that have garnered the interest of many researchers, this paper aims to conduct a bibliometric analysis of chitosan for sustainable development. Aranaz [1] reviewed chitosan for the green synthesis of metallic nanoparticles and biocatalysts. Salgado-Cruz [2] performed a bibliometric review on the application of chitosan for coating in postharvest products using the Scopus database for a period of 10 years. Kou et al. [4] reviewed ways to produce chitosan using chemical and biological methods. Maliki et al. [36] performed a minireview on chitosan for green applications. Hameed et al. [37] reviewed the applications of chitosan for filtration, metal removal, antibacterial properties, wound dressing, food preservation, agriculture, and drug delivery. Kostag and Seoud [38] reviewed the molecular structures of chitin and chitosan and studied the dissolution mechanism for these biopolymers. Klongthong [39] conducted a bibliometric analysis on the treatment of viral diseases with chitosan. Martău et al. [40] reviewed the applications of chitosan in the biomedical and food sectors. Ranjan [41] did a bibliometric analysis of the biomedical applications of chitosan. The top-cited paper by Crini [42] reviewed chitosan as one of the environmentally friendly absorbents that can be used for purification. The third-most-cited paper by Boateng et al. [43] reviewed wound-healing dressings using various polymers, including chitosan which helps in accelerating granulation.

Based on our search, a bibliometric analysis of chitosan for sustainable development has not been performed in past studies. Sustainable development is an important topic for the safety and wellbeing of all living organisms. Chitosan, which is a biopolymer, is cost effective with high sustainability and wide functionality. Therefore, this paper shall conduct a bibliometric analysis of chitosan for sustainable development using the Scopus database from 1976 to 2023 (as of 26 January 2023) [44,45,46]. A bibliometric analysis was conducted for performance analysis and scientific mapping of a research domain [47,48]. The outcome of a bibliometric analysis explains the development of the research domain over time. A bibliometric analysis also highlights the emerging trends in the domain to allow researchers to better identify the research gaps [49,50]. This analysis also aims to contribute by highlighting the sustainable applications of chitosan and pointing out the possible sustainable areas of research in chitosan. Section 2 explains the methodology used in this bibliometric analysis. Section 3 discusses the results of the bibliometric analysis. Section 4 concludes the paper with the highlights of the outcomes of the analysis.

## 2. Methodology

**Figure 1:**
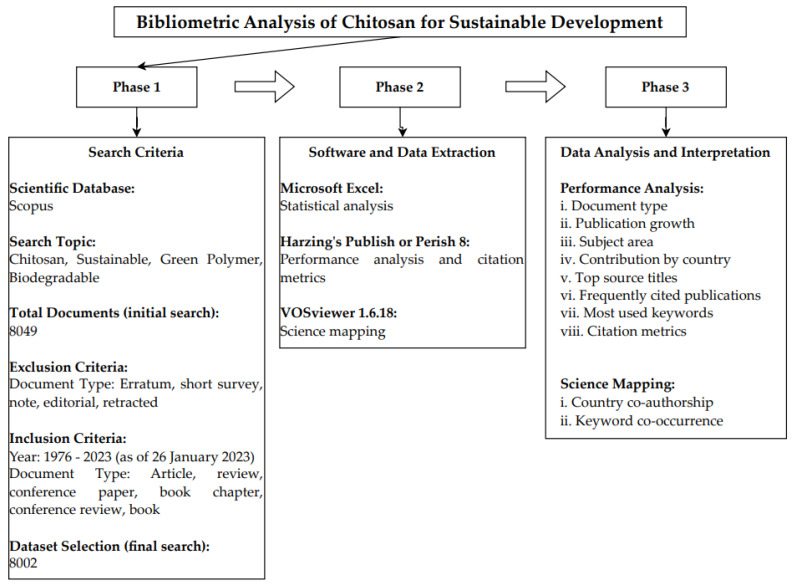
Process of bibliometric analysis of chitosan for sustainable development.

This paper intends to conduct a bibliometric analysis of chitosan for sustainable development. The search for scientific literature was performed on the Scopus database, which is a globally trusted database for high-quality peer-reviewed materials [51,52]. The Scopus database also has the most extensive coverage and widest citation records among all the scientific databases [53,54]. Figure 1 explains the process of the bibliometric analysis of chitosan for sustainable development [55,56].

In the first step, “chitosan” was identified as a biopolymer with wide applications and benefits to the present and future needs of the industry. Data were retrieved from the Scopus database on 26 January 2023. The following query was applied: TITLE-ABS-KEY (“chitosan” and (“sustainable*” or “green polymer” or “biodegradable*” or “ecofriendly*”)). From 1976 to 2023, 8049 documents were displayed. However, erratum, short survey, note, editorial, and retracted documents were excluded from this analysis [57,58,59]. A total of 8002 articles, reviews, conference papers, book chapters, conference reviews, and books were included in this bibliometric analysis.

In the second step, data were downloaded from the Scopus database for statistical analysis of bibliometric indicators. In the third step, further bibliometric analysis was conducted with Harzing’s Publish or Perish 8 and VOSviewer version 1.6.18. Harzing’s Publish or Perish was used for performance analyses with citation metrics for annual publication, documents by country, source titles, and highly cited documents [60]. In citation metrics, total document (TD) quantifies the number of documents, total citation (TC) shows the citations received for a document or author, *h*-index (*h* number of documents with minimum *h* number of citations) and *g*-index (*g* number of documents with minimum *g^2^* average citations) show the research achievements [61]. VOSviewer was used for scientific mapping, which maps the country coauthorship and keyword co-occurrence [46,62].

## 3. Results

The results of the bibliometric analysis of chitosan for sustainable development are discussed in this section. The results include document types, source types, annual publication, subject areas, country contribution, highly cited documents, and analysis of keywords. The summary of bibliometric analysis will be explained in the citation metrics for all 8002 documents from 1976 to 2023, as of 26 January 2023.

### 3.1. Document Type and Source Type

The 8002 documents are under six document types. Articles and reviews make up about 85% of the total documents. Of these, 5736 documents (71.68%) are articles while 1107 documents (13.83%) are reviews. Other document types are conference papers (560 documents or 7.00%), book chapters (509 documents or 6.36%), conference reviews (67 documents or 0.84%), and books (23 documents or 0.29%). Table 1 presents the document types of the 8002 papers on chitosan in sustainable development.

**Figure 2:**
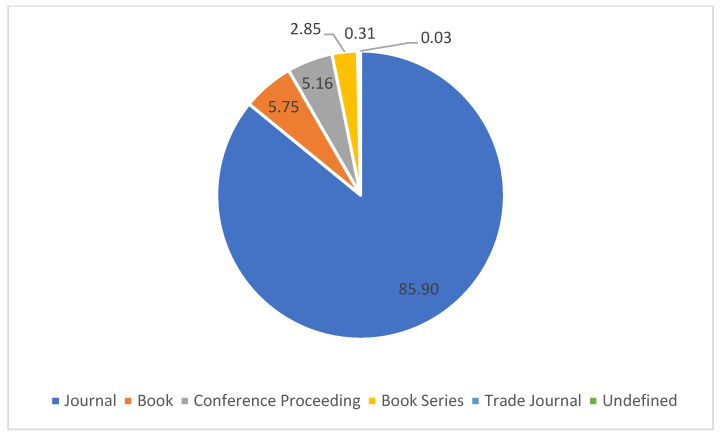
Source type.

Documents can be published in various sources, and 6874 documents (85.90%) were published in journals, 460 documents (5.75%) were published in books, 413 documents (5.16%) were published in conference proceedings, 228 documents (2.85%) were published in book series, while less than 0.35% of the documents were published in trade journals or were undefined. Figure 2 shows the source types of the documents on chitosan for sustainable development.

### 3.2. Annual Publication

The first paper listed on the Scopus database was published in 1976 and authored by Kohn [63]. This paper, titled “Shellfish wastes vie for cpi role”, noted that the shells of shrimps and crabs have chitin to derive chitosan, which is nontoxic and biodegradable. Chitosan can be used as flocculants, coagulants, food thickeners, or coatings, which are less toxic and less harmful than other chemicals [63]. This first paper has received two citations to date. The second and third papers listed on the Scopus database were published in 1986 by Koh et al. [64] and Machida et al. [65] which received 8 citations and 52 citations to date, respectively. The paper by Koh et al. [64] found that ground mixtures with chitosan offered a quicker dissolution rate of piroxicam than with chitin. Thus, chitosan can be used as a drug carrier. This study also noted that chitosan does not present a biological hazard. Machida et al. [65] performed an experiment to study the enzymatic degradation of chitosan and hydroxypropylchitosan on uracil. This paper then concluded that chitosan and hydroxypropylchitosan can be used in anticancer drugs.

**Figure 3:**
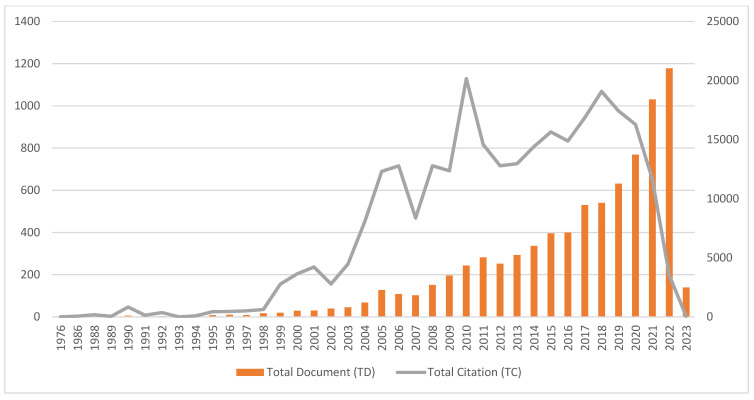
Total document and total citation.

Figure 3 describes the annual publication on chitosan for sustainable development and the total citation (TC) received by the documents. The focus on sustainability in chitosan was less prominent before the year 1995, as the total number of documents (TD) each year is below 10. An upward trend is observed from 1997 as the total number of documents (TD) peaked in 2022, with 1178 documents. As of 26 January 2023, 139 documents have been published and indexed in the Scopus database for the year 2023. This shows that researchers are increasingly interested in chitosan for sustainable development.

Table 2 presents the citation metrics of the annual publication of chitosan for sustainable development. The citation metrics include a number of cited documents (NCD), total citation (TC), citation per document (C/D), citation per cited document (C/CD), *h*-index, and *g*-index. The highest total citation was recorded in 2010 with 20,161 citations. This was largely contributed by the second and fourth highly cited papers by Kumari et al. [66] and Bhattarai et al. [67], respectively. Kumari et al. [66] received 2789 citations while Bhattarai et al. [67] received 1796 citations. The highest C/D and C/CD were recorded by the document published in 1988 by Hirano et al. [68]. This paper showed that chitosan can be used in oral and intravenous drug carriers.

### 3.3. Subject Area

In Scopus, documents are categorized under four main subjects including life sciences, physical sciences, health sciences, and social sciences and humanities. Then, there were 27 major subject areas and more than 300 minor subject areas. Every document may be categorized into more than one subject area based on Scopus classification [69,70]. The 8002 documents are grouped into several subject areas, with 18.56% of the documents grouped under materials science, followed by chemistry (14.46%), engineering (11.49%), chemical engineering (10.66%), and biochemistry, genetics, and molecular biology (10.18%). Pharmacology, toxicology, and pharmaceutics (6.33%), environmental science (5.23%), physics and astronomy (4.69%), agricultural and biological science (4.49%), and medicine (4.09%) are also under the top 10 subject areas. The complete list of subject areas is tabulated in Table 3.

### 3.4. Country Contribution

There were about 120 countries that contribute to the publication of chitosan on sustainable development. China (1560 documents), India (1400 documents), and the United States (800 documents) were the top three countries contributing to this domain. Iran, Brazil, Italy, South Korea, Egypt, Spain, and Malaysia also provided high contributions in this domain with 470, 347, 311, 305, 300, 269, and 251 documents, respectively. The United States received the highest total citation (TC) of 55,019, citation per document (C/D) of 68.77, and citation per cited document (C/CD) of 77.6%. The United States also had the highest publication impact with an *h*-index of 110 and a *g*-index of 211, and 110 documents published by researchers in the United States received at least 110 citations, respectively. At least 44,521 total citations were also received from 211 documents from the United States. Table 4 presents the top 10 country contribution.

**Figure 4:**
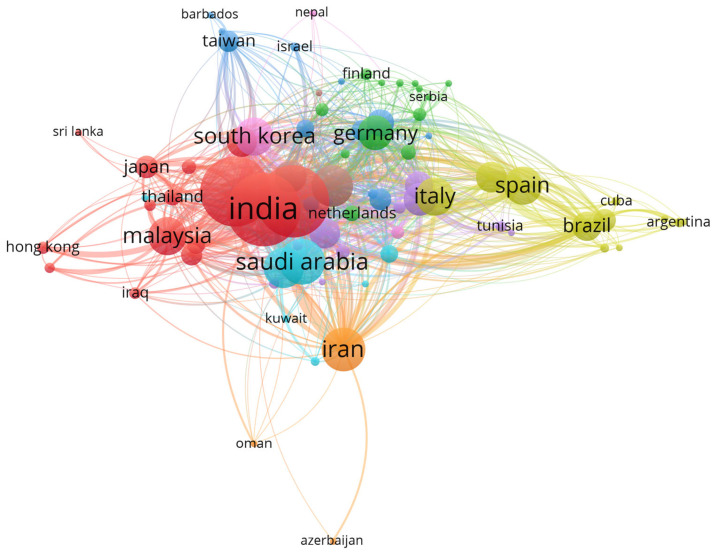
Country coauthorship.

VOSviewer maps the authors’ collaborations across countries. This feature allows a deeper understanding of the scientific collaboration among the authors in different countries [71]. Link strength explains the magnitude of collaboration. The larger the link strength, the higher the number of collaborations [72]. Table 5 shows the top 10 countries with the highest link strengths. India had the highest total link strength of 572 with 1400 total documents. This shows that India had the highest collaboration with authors from other countries. The United States had a 557 total link strength with 800 documents. Even though China had the highest document total of 1560 papers, China was only in the third position in international collaboration with a 527 total link strength. Saudi Arabia (263), Iran (248), the United Kingdom (228), Egypt (220), Italy (201), Malaysia (201), and South Korea (200) were also among the top 10 for total link strength in international author collaboration. Figure 4 maps the country’s coauthorship of the publications on chitosan for sustainable development.

Based on Figure 4, India has the largest node because of the highest total link strength. The thickest line spans from China to the United States with a link strength of 120. China and the United States collaborate the most in this domain. The colors of the nodes and lines represent the clustering of the countries [73]. There are nine clusters in total. The first cluster, which is red, has 15 countries made up of Australia, China, Hong Kong, India, Indonesia, Iraq, Japan, Malaysia, Nigeria, Singapore, South Africa, Sri Lanka, Thailand, the United States, and Vietnam. The second cluster, which is green, consists of Austria, Bangladesh, Bulgaria, Croatia, Finland, Germany, Hungary, Jordan, Lithuania, the Netherlands, Serbia, Slovenia, and Switzerland. The dark blue cluster consists of Barbados, Belgium, the Czech Republic, Denmark, Ethiopia, Israel, Latvia, the Philippines, Poland, the Russian Federation, Sweden, Taiwan, and Ukraine. The yellow cluster has Argentina, Brazil, Chile, Columbia, Cuba, Ecuador, Italy, Mexico, Peru, Portugal, Spain, and Venezuela. The next cluster, which is purple, is made up of Algeria, Canada, France, Greece, Lebanon, Morocco, Norway, Qatar, Romania, Tunisia, and Turkey. Egypt, Ireland, Kuwait, New Zealand, Saudi Arabia, Slovakia, and Yemen make up the light blue cluster. The orange cluster has Azerbaijan, Iran, and Oman. Kazakhstan, Pakistan, and the United Kingdom make up the brown cluster. The final cluster has Nepal, South Korea, and the United Arab Emirates.

### 3.5. Source Title

There were more than 150 source titles published on chitosan for sustainable development. Table 6 highlights the top 10 source titles. The source title that published the most documents on chitosan for sustainable development was “International Journal of Biological Macromolecules” (361), followed by “Carbohydrate Polymers” (271), “Polymers” (135), “Journal of Applied Polymer Science” (128), “Materials Science and Engineering C” (74), “Journal of Polymers and the Environment” (70), “Biomaterials” (69), “IOP Conference Series: Materials Science and Engineering” (69), “ACS Sustainable Chemistry and Engineering” (68) and “Molecules” (65). Documents published in “Biomaterials” received the highest total citation of 17,999. The top 10 source titles are also listed on Web of Science (WoS), except “Materials Science and Engineering C” and “IOP Conference Series: Materials Science and Engineering”.

### 3.6. Highly Cited Documents

Table 7 presents the top 10 highly cited documents on chitosan for sustainable development. The most-cited document, “Non-conventional low-cost adsorbents for dye removal: A review” by Crini [42], received 3590 citations. The review paper presented a critical analysis and the characteristics, advantages, limitations, and mechanisms of sorbents. Chitosan was identified as a promising adsorbent for environmental and purification purposes. The second-most-cited document, with 2789 citations, was by Kumari et al. [66] titled “Biodegradable polymeric nanoparticles based drug delivery systems”. The review paper discussed the impact of nanoencapsulation of various disease-related drugs on biodegradable nanoparticles such as chitosan and gelatin. The third-most-cited document, by Boateng et al. [43], discussed the common wound management dressings and novel polymers used for the delivery of drugs to various types of wounds. These included chitosan, hydrocolloids, hydrogels, alginates, collagen, polyurethane, hyaluronic acid, and pectin. The paper by Bhattarai et al. [67], titled “Chitosan-based hydrogels for controlled, localized drug delivery”, received 1796 citations and was the fourth highly-cited document. The authors investigated the developments in chitosan hydrogel preparation and defined the design parameters in the development of chemically and physically cross-linked hydrogels. The following most-cited paper by Khor and Lim [74] discussed the works of key groups in Asia developing chitosan and chitin materials for implantable biomedical applications.

Madihally and Matthew [75] authored the paper titled “Porous chitosan scaffolds for tissue engineering” which received 1338 citations. The authors studied the application of chitosan for the formation of porous scaffolds of controlled microstructures in tissue-relevant geometries. The seventh-most-cited paper by Chenite et al. [76] studied the use of polymer salt aqueous solutions as gelling systems and proposed the discovery of a prototype for a new family of thermosetting gels highly compatible with biological compounds. The following most-cited paper by Rieux et al. [77] discussed the influence of size and surface properties on the nanoparticles’ nonspecific uptake or their targeted uptake by enterocytes and M cells. Li et al. [78] obtained 1037 citations for their paper titled “Injectable and biodegradable hydrogels: gelation, biodegradation, and biomedical applications”. The authors presented the progress on biodegradable and injectable hydrogels fabricated from natural polymers such as chitosan and biodegradable synthetic polymers. The 10th-most-cited document was achieved by Klouda [79] for the paper “Thermoresponsive hydrogels in biomedical applications A seven-year update”. The author reviewed the literature on thermosensitive hydrogels by focusing on natural polymers as well as synthetic polymers.

### 3.7. Keyword Analysis

**Figure 5:**
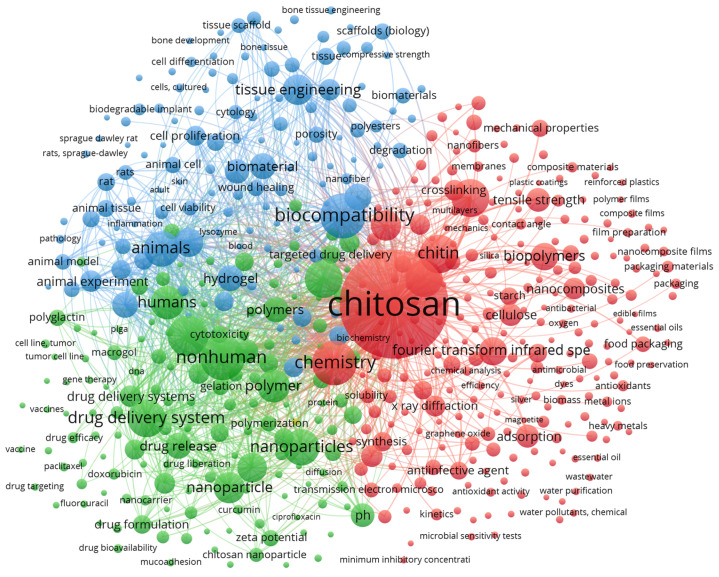
Keyword co-occurrence map.

VOSviewer provides a feature to map the keyword co-occurrence map to detect the research clusters and how these clusters are linked to form a subdomain [80]. Table 8 shows the 20 most frequently used keywords with the respective total link strengths. “Chitosan” (6201) was the most-used keyword with the highest total link strength of 93,328. The second-most-used keyword was “biocompatibility” with 1525 occurrences. The third-most-used keyword was “nonhuman” with 1492 occurrences. Figure 5 depicts the keyword co-occurrence map.

Based on Figure 5, the keywords can be categorized into three clusters. The first cluster is red and contains keywords such as acetylation, antibacterial activity, antiinfective agent, antimicrobial activity, antioxidants, bioactivity, biodegradable, biodegradation, biopolymer, bioremediation, carbon dioxide, carboxymethyl chitosan, catalysis, cellulose, chitin, chitosan, chitosan derivatives, chlorine compounds, coagulation, coating, crystallinity, deacetylation, differential scanning calorime, ecofriendly, electrolytes, environmental impact, *Escherichia coli*, ethylene, flocculation, food packaging, functional polymers, glycerol, green chemistry, heavy metals, hydrogen bond, hydrolysis, II-VI semiconductors, infrared spectroscopy, ions, lignin, microbial sensitivity test, minimum inhibitory concentration, morphology, nanofiber, plant extract, polysaccharides, temperature, tensil strength, thermodynamics, ultraviolet radiation, wastewater treatment, water vapor permeability, wound dressings, and X-ray diffraction.

The second cluster, which is green, contains alginates, antineoplastic agents, apoptosis, bovine serum albumin, cancer therapy, chondroitin sulfate, cyclodextrin, cytotoxicity, DNA, drug carrier, drug delivery, emulsion, encapsulation, gel, gelatin, gene therapy, hexuronic acids, human, hyaluronic acid, hydrophilicity, hydrophobicity, immunogenicity, liposome, macrogol, molecular structure, nanotechnology, nonhuman, paclitaxel, pH, polyethyleneimine, polyvinyl alcohol, vaccine, and zeta potential. The third cluster is blue and has adhesion, animal, angiogenesis, biomimetics, bone regeneration, calcium phosphate, cartilage, cell proliferation, cytology, fibroblast, flow kinetics, freeze drying, hydrogel, in vitro study, in vivo study, lysozyme, pharmacology, scaffold, tissue engineering, and Young modulus.

**Figure 6:**
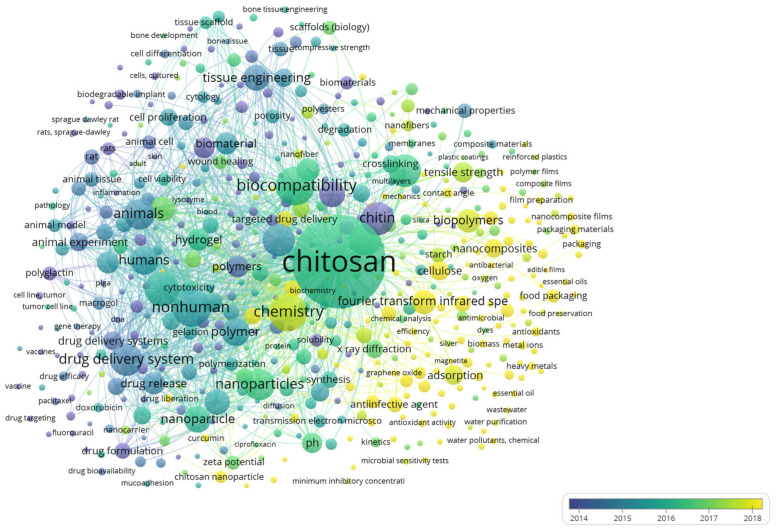
Keyword overlay visualization map.

Figure 6 shows the overlay visualization map of keywords, which explains the trends of the publications on chitosan for sustainable development. The yellow keywords are the recent trends and the current focus of researchers. They include sustainability, graphene oxide, antibacterial agents, field emission scanning electron microscopy, *Staphylococcus aureus*, *Pseudomonas aeruginosa*, water management, water pollutant, heavy metal, recycling, food packaging, *Escherichia coli*, lignin, contact angle, II-VI semiconductors, and microbial sensitivity test.

Green electronics are also significant in sustainable development. Based on our query, there were several papers that adopt chitosan in green electronics and are impactful to current and future research. Chitosan and polyvinylpyrrolidone substrates produced using a solution casting process have great optical transmittance, high temperature stability, high biodegradation rate, and excellent mechanical stability to be used in flexible electronics [81]. Miao et al. [32] have also experimented with a starch–chitosan substrate for wearable green electronics. Du et al. [35] used chitosan with Y_2_O_3_ in organic thin-film transistors with superior dielectric properties. Li et al. [82] performed a review on the use of chitosan in electronic devices such as solar cells, organic field-effect transistors, and light-emitting diodes (LED). In addition, chitosan-based solid carbon dots can also be used for white LED and 3D printing [83]. Chitosan-mediated LED illumination also has better antibacterial treatment on *Escherichia coli*, *Listeria monocytogenes*, and *Salmonella* spp. than LED illumination only [84]. Chitosan nanoparticles with LEDs of different spectra can also modify *Eleutherococcus senticosus* for the treatment of diseases [85]. Chitosan-based asymmetric electrodes also have high selectivity and absorption of oxidized hexavalent uranium, strontium cation, and cesium [86]. Therefore, the applications of chitosan in green electronics are beneficial in food science, nutrition, medicine, technology, and water treatment. Chitosan is also the current trend based on the keyword overlay visualization.

The research on water purification and the management of water pollutants are insightful areas with broad prospects. There are more than 100 articles discussing the removal of impurities and purification. Liu et al. [87] experimented with the usage of chitosan-modified cellulose fibers and ferric chloride to remove *Microcystis aeruginosa* and microcystin-LR. In their book, Ahankari et al. [88] studied water remediation and purification and found that nanochitosan-based materials have a better absorption capacity than microsized chitosan when removing heavy metals due to a larger surface area and reactivity. Magnetic chitosan nanoparticles also have great superparamagnetism and are low cost with high efficiency [88]. Chitosan-AgIO_3_ can also treat *Pseudomonas aeruginosa*, *Klebsiella pneumoniae*, *Staphylococcus saprophyticus*, *Escherichia coli*, and *Staphylococcus aureus* which are helpful in water purification [89]. Photothermal chitosan–cellulose nanofiber is also a promising solar-driven cost-effective water purification technique [90]. Water treatment and heavy metal removal for purification have also been studied in various pieces of research [91,92,93,94,95,96,97].

However, there are also some challenges to using chitosan. Mujtaba et al. [98] stated that chitosan has low hydrophobicity and low mechanical and thermal strength which inhibits its use as a barrier enhancer in food packaging. Therefore, chitosan has been combined with other biopolymers, plant or animal proteins, waxes, and minerals while other methods such as cross-linking, graft copolymerization, and enzymatic treatments are used to improve the barrier properties of chitosan. Meanwhile, the stability of chitosan is also a challenge in pharmaceutical and biomedical fields [99]. The purity of chitosan affects the drug delivery process. Chitosan may not be able to dissolve due to the presence of ash or residual proteins for drug delivery [100,101,102]. Chitosan may also degrade through enzymatic hydrolysis if there is microbiological contamination. Centrifugation and extensive shearing could also reduce the molecular weight of chitosan and cause fluctuation in the polydisperity index in biomedical applications [103,104]. A low degree of deacetylation (DD) also causes acute inflammation and low affinity to enzymes in vitro. On the other hand, chitosan with high DD is less porous with low water uptake which slows down the rate of acidic hydrolysis [105,106].

During storage, the physiochemical and mechanical characteristics of chitosan may be altered due to the change in moisture level. Prolonged storage can dehydrate chitosan which then reduces its crushing strength and causes a spike in friability and disintegration [107]. Excessive moisture in the chitosan structure will increase the damage level due to hydrolysis. When the storage environment has a high relative humidity, the mechanical properties of chitosan will be decreased, as there is more swelling of the chitosan to induce a quicker release of the active compounds [108]. This will also bring down the adhesiveness of chitosan carriers with mucin [109,110,111]. The application of chitosan in hydrogels may be unstable as dissolution may happen, has poor mechanical resistance, and its pore size is hard to control, while chemical crosslinking may alter its intrinsic properties. Chitosan sponge may shrivel and is less porous [112]. Since there is vast potential to use chitosan, especially in the pharmaceutical and biomedical industries, researchers may identify these suitable research gaps, which may be studied to overcome the limitations of chitosan.

### 3.8. Citation Metric

The citation metric of the 8002 documents on chitosan for sustainable development from 1976 to 2023 as of 26 January 2023 is shown in Table 9. Based on the 8002 documents, 278,578 citations have been received with an *h*-index of 215 and a *g*-index of 343.

## 4. Conclusions

The research on chitosan for sustainability has received notable attention in recent years. This can be seen in the upward trend of annual publications where the number of documents exceeded 1000 in the year 2021 and continued to rise in 2022. Most of the documents were articles published in journals. The first paper indexed in Scopus was “Shellfish wastes vie for cpi role” by Kohn [63] which was published in 1976. The highest cited document is “Non-conventional low-cost adsorbents for dye removal: A review” by Crini [42] which received 3590 citations since its publication in 2006. China (1560) produced the highest number of documents on chitosan for sustainable development. The United States received the highest total citation of 55,019, 68.77 citations per document and 77.6 citations per cited document. The top three source titles which publish documents on chitosan for sustainable development were the “International Journal of Biological Macromolecules”, “Carbohydrate Polymer” and “Polymers”. All these three source titles were also indexed in WoS.

Most of the documents were under materials science, chemistry, and engineering. India had the highest international collaboration with 572 total link strength on the 1400 documents. The biggest link strength (120) was found between China and the United States. From the keyword co-occurrence map, it can be highlighted that the research trend is moving toward the application of chitosan for sustainable development. Increased research interest has been placed on the antibacterial functionality of chitosan on bacteria such as *staphylococcus aureus*, *pseudomonas aeruginosa*, and *Escherichia coli*. Lately, chitosan has also been studied for its flocculation in water treatment to remove organic matter, suspended solids, and heavy ions for a sustained environment.

There are several limitations to this study. Firstly, the Scopus database is constantly updating from time to time. Therefore, this bibliometric analysis may be repeated in the future for an intensive understanding of the evolving trends. Secondly, the first indexed paper in the Scopus database was published in 1976. Documents published before 1976 which were not indexed in the Scopus database were not considered in this study.

## Figures and Tables

**Table 1:** Document type.

Document Type	Total Documents	Percentage (%)
Article	5736	71.68
Review	1107	13.83
Conference Paper	560	7.00
Book Chapter	509	6.36
Conference Review	67	0.84
Book	23	0.29
**Total**	**8002**	**100**

**Table 2:** Annual Publication.

Year	TD ^1^	Percentage (%)	Cumulative Percentage (%)	NCD ^2^	TC ^3^	C/D ^4^	C/CD ^5^	*h*-Index	*g*-Index
1976	1	0.01	0.01	1	2	2	2	1	1
1986	2	0.02	0.04	2	60	30	30	2	2
1988	1	0.01	0.05	1	175	175	175	1	1
1989	2	0.02	0.07	2	48	24	24	2	2
1990	6	0.07	0.15	5	830	138.33	166	3	6
1991	4	0.05	0.20	4	142	35.5	35.5	4	4
1992	3	0.04	0.24	3	363	121	121	3	3
1993	2	0.02	0.26	0	0	0	0	0	0
1994	3	0.04	0.30	3	90	30	30	3	3
1995	8	0.10	0.40	5	439	54.88	87.8	5	8
1996	10	0.12	0.52	9	451	45.1	50.11	7	10
1997	8	0.10	0.62	8	509	63.63	63.63	5	8
1998	17	0.21	0.84	16	620	36.47	38.75	9	17
1999	20	0.25	1.09	18	2774	138.7	154.11	14	20
2000	29	0.36	1.45	28	3642	125.59	130.07	21	29
2001	30	0.37	1.82	29	4219	140.63	145.48	24	30
2002	39	0.49	2.31	36	2787	71.46	77.42	24	39
2003	45	0.56	2.87	44	4469	99.31	101.57	24	45
2004	68	0.85	3.72	62	8086	118.91	130.42	38	68
2005	127	1.59	5.31	109	12,313	96.95	112.96	52	110
2006	108	1.35	6.66	96	12,771	118.25	133.03	47	108
2007	102	1.27	7.94	95	8362	81.98	88.02	45	91
2008	151	1.89	9.82	137	12,787	84.68	93.34	53	112
2009	196	2.45	12.27	182	12,359	63.06	67.91	60	107
2010	243	3.04	15.31	229	20,161	82.97	88.04	62	139
2011	282	3.52	18.83	256	14,576	51.69	56.94	67	113
2012	252	3.15	21.98	227	12,785	50.73	56.32	59	106
2013	293	3.66	25.64	262	12,965	44.25	49.48	61	105
2014	336	4.20	29.84	301	14,408	42.88	47.87	60	107
2015	396	4.95	34.79	363	15,649	39.52	43.11	64	109
2016	400	5.00	39.79	368	14,882	37.21	40.44	62	104
2017	530	6.62	46.41	458	16,842	31.78	36.77	63	107
2018	540	6.75	53.16	508	19,074	35.32	37.55	66	110
2019	631	7.89	61.05	591	17,423	27.61	29.48	63	96
2020	769	9.61	70.66	709	16,277	21.17	22.96	59	86
2021	1031	12.88	83.54	887	11,654	11.3	13.14	43	61
2022	1178	14.72	98.26	725	3541	3.01	4.88	22	31
2023	139	1.74	100.00	20	43	0.31	2.15	3	5
Total	8002	100			278,578				

^1^ Total Document; ^2^ Number of Cited Documents; ^3^ Total Citation; ^4^ Citations per Document; ^5^ Citations per Cited Document.

**Table 3:** Subject Area.

Subject Area	Total Document	Percentage (%)
Materials Science	3249	18.56
Chemistry	2531	14.46
Engineering	2010	11.49
Chemical Engineering	1865	10.66
Biochemistry, Genetics, and Molecular Biology	1782	10.18
Pharmacology, Toxicology, and Pharmaceutics	1108	6.33
Environmental Science	915	5.23
Physics and Astronomy	821	4.69
Agricultural and Biological Sciences	786	4.49
Medicine	716	4.09
Energy	540	3.09
Economics, Econometrics, and Finance	276	1.58
Immunology and Microbiology	234	1.34
Computer Science	131	0.75
Business, Management, and Accounting	100	0.57
Multidisciplinary	91	0.52
Earth and Planetary Sciences	90	0.51
Social Sciences	56	0.32
Health Professions	49	0.28
Veterinary	34	0.19
Mathematics	32	0.18
Neuroscience	30	0.17
Dentistry	21	0.12
Nursing	20	0.11
Arts and Humanities	7	0.04
Psychology	4	0.02
Decision Sciences	3	0.02
Total	17,501	100

**Table 4:** Country Contribution.

Country	TD ^1^	NCD ^2^	TC ^3^	C/D ^4^	C/CD ^5^	*h*-Index	*g*-Index
China	1560	1354	50,477	32.36	37.28	100	163
India	1400	1197	48,529	34.66	40.54	101	183
United States	800	709	55,019	68.77	77.6	110	211
Iran	470	402	13,940	29.66	34.68	60	98
Brazil	347	307	8471	24.41	27.59	48	78
Italy	311	277	10,406	33.46	37.57	51	92
South Korea	305	267	10,931	35.84	40.94	49	95
Egypt	300	256	8301	27.67	32.43	46	83
Spain	269	242	11,411	42.42	47.15	49	100
Malaysia	251	212	5487	21.77	25.88	39	66

^1^ Total Document; ^2^ Number of Cited Documents; ^3^ Total Citations; ^4^ Citations per Document; ^5^ Citations per Cited Document.

**Table 5:** Top 10 countries for author collaborations.

Country	Total Document	Total Link Strength
India	1400	572
United States	800	557
China	1560	527
Saudi Arabia	164	263
Iran	470	248
United Kingdom	204	228
Egypt	300	220
Italy	311	201
Malaysia	251	201
South Korea	305	200

**Table 6:** Source Title.

Source Title	TD ^1^	Percentage (%)	TC ^2^	Publisher	Cite Score	SJR ^3^	SNIP ^4^	*h*-Index	JIF ^5^	JCI ^6^
International Journal of Biological Macromolecules	361	4.51	16,073	Elsevier	11.6	1.100	1.449	144	8.025	1.42
Carbohydrate Polymers	271	3.39	14,519	Elsevier	16.0	1.612	1.821	228	10.723	2.19
Polymers	135	1.69	2286	Multidisciplinary Digital Publishing Institute (MDPI)	5.7	0.726	1.170	89	4.967	0.88
Journal Of Applied Polymer Science	128	1.60	2968	Wiley-Blackwell	5.0	0.528	0.793	175	3.057	0.61
Materials Science and Engineering C	74	0.92	4613	Elsevier	12.6	1.191	1.417	145	NIL ^7^	NIL ^7^
Journal Of Polymers and The Environment	70	0.87	1411	Springer Nature	6.8	0.648	1.038	80	4.705	0.65
Biomaterials	69	0.86	17,999	Elsevier	21.5	2.678	2.045	397	15.304	2.68
IOP Conference Series: Materials Science and Engineering	69	0.86	138	IOP Publishing Ltd.	1.1	0.249	0.344	48	NIL ^7^	NIL ^7^
ACS Sustainable Chemistry and Engineering	68	0.85	2513	American Chemical Society	14.5	1.743	1.361	132	9.224	1.44
Molecules	65	0.81	1822	Multidisciplinary Digital Publishing Institute (MDPI)	5.9	0.705	1.267	171	4.927	0.64

^1^ Total Document; ^2^ Total Citation; ^3^ SCImago Journal Rank 2021; ^4^ Source Normalized Impact per Paper 2021; ^5^ Journal Impact Factor 2021; ^6^ Journal Citation Indicator 2021, ^7^ Data Not Available.

**Table 7:** Highly Cited Documents.

Author	Title	Year	Cites	Cites Per Year	Source Title
G. Crini [42]	Non-conventional low-cost adsorbents for dye removal: A review	2006	3590	211.18	Bioresource Technology
A. Kumari, S.K. Yadav, S.C. Yadav [66]	Biodegradable polymeric nanoparticles based drug delivery systems	2010	2789	214.54	Colloids and Surfaces B: Biointerfaces
J.S. Boateng, K.H. Matthews, H.N.E. Stevens, G.M. Eccleston [43]	Wound healing dressings and drug delivery systems: A review	2008	1945	129.67	Journal of Pharmaceutical Sciences
N. Bhattarai, J. Gunn, M. Zhang [67]	Chitosan-based hydrogels for controlled, localized drug delivery	2010	1796	138.15	Advanced Drug Delivery Reviews
E. Khor, L.Y. Lim [74]	Implantable applications of chitin and chitosan	2003	1468	73.4	Biomaterials
S.V. Madihally, H.W.T. Matthew [75]	Porous chitosan scaffolds for tissue engineering	1999	1338	55.75	Biomaterials
A. Chenite, C. Chaput, D. Wang, C. Combes, M.D. Buschmann, C.D. Hoemann, J.C. Leroux, B.L. Atkinson, F. Binette, A. Selmani [76]	Novel injectable neutral solutions of chitosan form biodegradable gels in situ	2000	1199	52.13	Biomaterials
A. des Rieux, V. Fievez, M. Garinot, Y.-J. Schneider, V. Préat [77]	Nanoparticles as potential oral delivery systems of proteins and vaccines: A mechanistic approach	2006	1068	62.82	Journal of Controlled Release
Y. Li, J. Rodrigues, H. Tomás [78]	Injectable and biodegradable hydrogels: Gelation, biodegradation and biomedical applications	2012	1037	94.27	Chemical Society Reviews
L. Klouda [79]	Thermoresponsive hydrogels in biomedical applications	2008	989	65.93	European Journal of Pharmaceutics and Biopharmaceutics

**Table 8:** Top 20 Keywords with Total Link Strengths.

Keywords	TD ^1^	Total Link Strength
Chitosan	6201	93,328
Nonhuman	1492	38,976
Chemistry	1468	37,602
Human	1287	34,247
Biocompatibility	1525	31,074
Drug delivery system	1143	28,912
Animals	1051	28,575
Humans	944	26,310
Biodegradability	1122	23,721
Scanning electron microscopy	1103	23,341
Nanoparticles	1060	21,317
Polymer	900	21,023
Animal	750	20,874
Nanoparticle	820	20,062
Particle size	767	19,536
Tissue Engineering	795	19,081
In vitro study	620	18,166
Chitin	1163	17,624
Biodegradable polymers	1146	17,262
Biomaterial	611	16,379

^1^ Total Document.

**Table 9:** Citation Metric.

Items	Metrics
Date of Extraction	26 January 2023
Papers	8002
Citations	278,578
Years	47
Citation per Year	5927.19
Citation per Document	34.81
Citation per Author	80,267.14
Papers per Author	2135.23
Authors per Paper	4.86
*h*-index	215
*g*-index	343

## Data Availability

The data presented in this study are available on request from the corresponding author.
